# Microvillous cells in the olfactory epithelium express elements of the solitary chemosensory cell transduction signaling cascade

**DOI:** 10.1371/journal.pone.0202754

**Published:** 2018-09-13

**Authors:** Federica Genovese, Marco Tizzano

**Affiliations:** Monell Chemical Senses Center, Philadelphia, Pennsylvania, United States of America; Duke University, UNITED STATES

## Abstract

The nasal cavity hosts an array of chemoresponsive cells, including the extended olfactory system and several other cells involved in detection of and responses to irritants. Solitary chemosensory cells (SCCs), which respond to irritants and bacteria, express the transient receptor potential channel TRPM5 an essential element of the taste transduction-signaling cascade. Microvillous cells (MVCs), non-neuronal cells situated in the apical layer of the main olfactory epithelium, also express TRPM5, but their function has not yet been clarified. TRPM5-positive MVCs, like SCCs, show a cholinergic phenotype expressing choline acetyl transferase (ChAT), but none of the other elements of the bitter taste transduction cascade could be detected. We reexamined TRPM5-positive MVCs with more sensitive gene expression and staining techniques to clarify whether they rely only on TRPM5 and ChAT or express other elements of the taste/SCC transduction cascade. Analyzing existing RNA sequencing data from whole olfactory mucosa and isolated olfactory sensory neurons, we determined that several elements of the taste/SCC transduction cascade, including taste receptors, are expressed in the olfactory mucosa in cells other than olfactory sensory neurons. Immunostaining confirmed the presence TRPM5 and ChAT in a subset of cells of the olfactory mucosa, which also showed the expression of PLCB2, gustducin, and T1R3. Specifically, these cells were identified as TRPM5-positive MVCs. Furthermore, we examined whether MVCs are innervated by trigeminal fibers, similarly to SCCs. Using antibodies against trigeminal nerve markers calcitonin gene-related peptide and substance P, we determined that, despite the cholinergic phenotype, most MVCs in the olfactory mucosa lacked consistent trigeminal innervation. Our findings indicate that MVCs, like SCCs, express all the elements of the bitter taste transduction cascade but that, unlike SCCs, they possess only sparse trigeminal innervation. The cholinergic phenotype of MVCs suggests a modulatory function of the surrounding olfactory epithelium, through the release of acetylcholine.

## Introduction

In the last few decades, a wide range of different chemoresponsive cells have been described in rodents, including olfactory sensory neurons (OSNs), taste receptor cells, vomeronasal organ neurons, and trigeminal neurons, responsible, respectively, for the detection of odors, tastants, pheromones, and noxious stimuli [1–7]. More recently, another chemoresponsive population of cells, the solitary chemosensory cells (SCCs), has been described in the nasal respiratory epithelium (RE) and vomeronasal organ ducts of rodents [8,9]. SCCs express molecular markers of the taste transduction signaling cascade, including taste receptors (T1Rs and T2Rs), transient receptor potential channel 5 (TRPM5), the G protein α-gustducin, phospholipase C beta 2 (PLCB2) and the inositol 1,4,5-trisphosphate receptor, type 3 [10,11]. These cells respond to a wide variety of chemicals, including bitter compounds, odorants, and bacterial signaling molecules [12–14]. Once activated, SCCs release acetylcholine (ACh) at the level of the cholinergic synapsis with the peptidergic trigeminal sensory fibers (immunoreactive to calcitonin gene-related peptide [CGRP] and substance P [SubP]), triggering trigeminal-mediated protective reflexes, such as apnea or sneezing, which are associated with local inflammation [9,12,13,15,16]. Although SCCs were initially identified in the RE and vomeronasal organ, TRPM5-expressing cells were also reported in the main olfactory epithelium (MOE), including a small subset of OSNs [17] and the microvillous cells (MVCs) [18,19].

**Fig 1:**
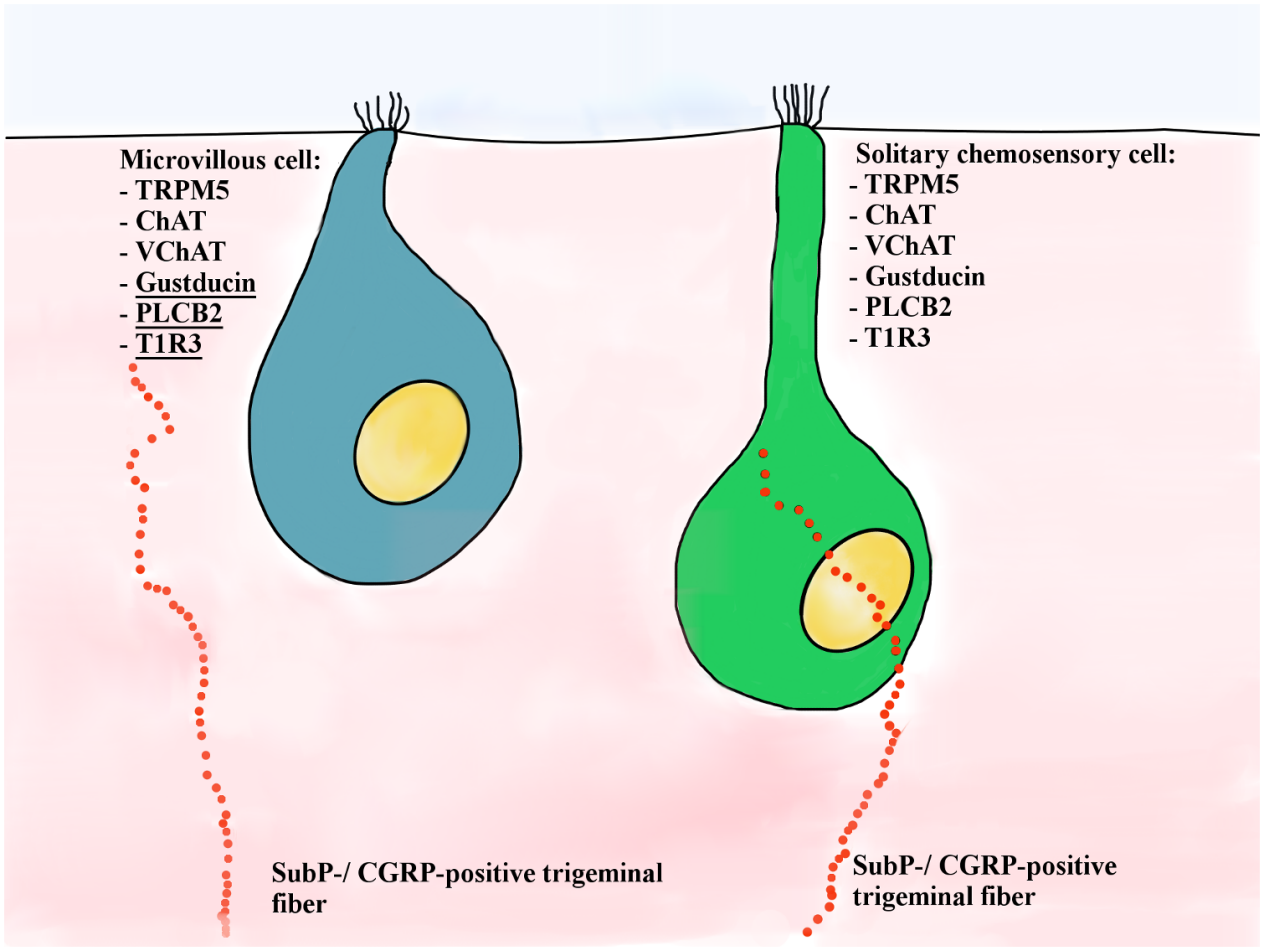
Schematic representation of TRPM5-positive MVCs and SCCs. Previously known molecular markers and innervation pattern are represented. We report here the presence in MVCs of other SCC transduction signaling cascade elements: gustducin, PLCβ2, and T1R3 (underlined). Other than a few morphological differences and the fact that MVCs are not innervated by peptidergic trigeminal nerve fibers, SCCs and MVCs are the same cell type in two different locations of the nasal cavity.

TRPM5-positive MVCs are small epithelial cells located above the layer of OSNs and supporting/sustentacular cells [18]. Based on their morphology, TRPM5-positive MVCs were classified as type a and type b. Type a MVCs are medium-sized cells located approximately 20 μm from the surface of the epithelium, with a slightly elevated apex and “stiff” microvilli radiating from the top. Type b MVCs were observed in the uppermost 10 μm of the MOE, with a “pear-shaped” cell body, flat apex, and microvilli slightly shorter and thinner than observed in type a MVCs [18]. Morphology aside, type a and b MVCs share a protein expression profile similar to that of SCCs, such as TRPM5, choline acetyltransferase (ChAT), and vesicular acetylcholine transporter (VAChT), but not all the other elements of the bitter taste transduction signaling cascade (Fig 1) [16,20].

In this study we analyzed RNA sequencing (RNA-seq) data obtained by Saraiva [21] to determine the expression levels of different elements of the bitter taste signaling transduction cascade and used immunohistochemistry to show their expression pattern in the olfactory epithelium. The RNA-seq data set shows that differential expression values for OSN markers in the isolated neuron samples are at least 3-fold higher than expression in whole olfactory mucosa (WOM) samples. On the other hand, higher expression in the WOM than in the OSNs (starting from a fold change of 0.33 or lower) indicates that the gene is likely not expressed in the OSNs but instead is expressed in other WOM cell types [21]. Furthermore, the OSN/WOM expression ratio suggests that these genes are mainly expressed in cell types different from the OSNs. Our analyses of these existing RNA-seq data provide both a quantitative characterization of the single gene expression and indicate which cell type likely expresses it.

Driven by the RNA-seq data and to determine which WOM cell types express genes of interest, we performed an immunohistochemical analysis of the taste transduction cascade protein expression: the taste receptor T1R3, the G protein α-gustducin, phospholipase C beta2 (PLCβ2), and other elements of the taste transduction signaling cascade. Previous immunohistochemical attempts were unsuccessful to detect other elements of the taste or olfactory transduction cascades in MVCs [18,19]. Using an immunohistochemical staining technique that requires parafolmaldehyde/lysine/periodate (PLP), we were able to detect T1R3, gustducin, and PLCβ2 in TRPM5-positive or ChAT-positive MVCs in the MOE, in contrast to previous results [18,19,22]. Therefore, like SCCs in the RE, MVCs in the MOE express many elements of the bitter taste transduction signaling cascade, while they do not express the olfactory marker protein (OMP), the ubiquitin carboxyl-terminal hydrolase PGP9.5, the cyclic nucleotide gated olfactory channel (CNG2A) and adenylyl cyclase III (ACIII), proteins or olfactory transduction factors known to be expressed specifically in OSNs [19]. As previously reported, the MOE is also characterized by the presence of trigeminal peptidergic fibers containing SubP and CGRP [7,13,23]. In contrast to SCCs in the RE and vomeronasal organ, we could not detect any significant synaptic contact between MVCs and SubP- or CGRP-positive peptidergic trigeminal fibers in the MOE. Thus, we can conclude that MVCs are molecularly and functionally almost identical to SCCs but that their action might be limited to the protection of the local olfactory epithelium, whereas SCCs are involved in the protection of the entire upper airways.

## Materials and methods

### Animals

All experimental procedures followed the National Institutes of Health guidelines for the care and use of animals and were in compliance with the guidelines of and approved by the Monell Chemical Senses Center Animal Care and Use Committee. In order to characterize MVCs in the MOE, we used four genetically modified transgenic mouse lines, each of them expressing green fluorescent protein (GFP) under the promoter for *TrpM5*, *Tas1R3*, *Plcb2* or *Chat*, respectively [24–26]. TrpM5-GFP mice contain a TrpM5-GFP construct, including 11 kb of mouse *TrpM5* 5′ flanking sequence, *TrpM5* exon 1 (untranslated), intron 1, and the untranslated part of exon 2, and enhanced-GFP (eGFP) [26]. The Tas1R3-GFP construct contained from 5′ to 3′: 13 kb of the mouse *Tas1R3* gene, including the 5′ flanking region and the entire 5′ untranslated region, and the coding sequence for eGFP [26]. The Chat-GFP mouse expresses tau-protein-GFP (inserted inside the third exon) driven by the promoter for *Chat* [24]. Wild-type (C57BL/6), TrpM5-GFP, TrpM5-GFP/TrpM5 knockout (KO), Tas1R3-GFP, and Chat-GFP mice were bred and housed in the animal facility of the Monell Chemical Senses Center. The Plcb2-GFP mice, kindly provided by Dr. Nirupa Chaudhari, were bred and housed in the animal facilities of the University of Miami (Miami, FL). In this transgenic mouse line GFP is expressed under the control of the *Plcb2* promoter [25]. Polymerase chain reaction was used to genotype the offspring of these strains to confirm the presence of GFP.

### Immunohistochemistry

Wild-type (C57BL/6), TrpM5-GFP, TrpM5-GFP/TrpM5-KO, Tas1R3-GFP, and Chat-GFP mice were used. The animals were housed in a pathogen-free environment under standardized conditions. Food and water were provided ad libitum. For each line, five mice of both sexes aged between 2 weeks and 6 months were anesthetized with pentobarbital (100 mg/kg), perfused transcardially with 0.9% saline followed by paraformaldehyde/lysine/periodate (PLP) fixation solution (1.6% paraformaldehyde, 75 mM lysine, 10 mM sodium periodate, pH 7.2). The olfactory organs were dissected and postfixed in the same fixative for 1 hour to overnight. Cryoprotection was carried out in 30% sucrose overnight. The tissue was embedded in Tissue Tek OCT (Sakura Finetek, Torrance, CA). Cryosections (12–14 μm) were mounted on Superfrost Plus slides (VWR, West Chester, PA) and frozen at -80°C until further use. Standard immunohistochemical procedures were used [14]. Briefly, cryosections were washed in 0.1 M phosphate-buffered saline (PBS), blocked in blocking solution containing 1% bovine serum albumin, 3–5% normal serum, and 0.3% Triton X-100 in PBS for 2 hours and then incubated in the primary antisera overnight to 3 days. For details of antibodies see Table 1. After three washes with PBS, 20 min each, the sections were incubated in the appropriate secondary antibodies (Alexa 488, Alexa 568, 1:400; Invitrogen, Carlsbad, CA, DL549, DL649, Jackson ImmunoResearch, West Grove, PA) for 2 hours at room temperature. After incubation with secondary antibodies, sections were washed three times, 20 min each, and coverslipped with Fluormount-G (Fisher Biotech, Birmingham, AL). Control slides were treated either without the primary antibody or with normal rabbit serum replacing the primary antiserum. When the appropriate peptides were available, the antisera were adsorbed with the blocking peptides. Control sections showed no labeling. The antiserum against TRPM5 when tested in TrpM5-GFP/TrpM5-KO mice showed no label in the MOE sections confirming the specificity of the TRPM5 antibody in this tissue. Images of the sections of interest were acquired using a fluorescence microscope (Nikon Eclipse 80i) or a confocal laser microscope (Leica DMRXE). Pictures were processed in Adobe Photoshop, version CS2 (Adobe Systems Inc., San Jose, CA).

**Table 1:** Antisera used to characterize TRPM5-positive MV cells.

Antisera against	Marker for	Company	Lot	Host; Concentration
Adenylate cyclase 3 (ACIII)	Olfactory transduction cascade: cilia OSNs	Santa Cruz sc-588	J3009	rabbit; 1:100
Calcitonin gene-related peptide (CGRP)	Peptidergic trigeminal nerve fibers	Peninsula Lab. T-4032	040826–4	rabbit; 1:2,000
Calcitonin gene-related peptide (CGRP)	Peptidergic trigeminal nerve fibers	Abcam AB36001	833910	goat; 1:200
Choline acetyltransferase (ChAT)	Synthesis of ACh	Chemicon/Millipore AB144P	LV1443701 LV1580976 JC161887	goat; 1:200
Cyclic nucleotide-gated channel alpha-2 (CNGA2)	Olfactory transduction cascade: cilia OSNs	Santa Cruz sc-13700	—	goat; 1:100
Green fluorescent protein (GFP)	—	Aves GFP-1020	1229FP08	chicken; 1:2000
G-protein subunit alpha-3 (gustducin)	Taste transduction cascade: SSCs and MVCs	Santa Cruz sc-395	D0808, D181, E1704	rabbit; 1:500, 1:1,000
Olfactory marker protein (OMP)	OSNs	Dr. F. Margolis, University of Maryland	—	goat; 1:5000
Protein gene product 9.5 (PGP 9.5)	OSNs	AbD Serotec 7663–0504	071207 230109	rabbit; 1:1000
Phospholipase C beta-2 (PLCβ2)	Taste transduction cascade: SCCs and MVCs	Santa Cruz Sc-206	I181, A1204, B0907	rabbit; 1:500
Substance P (SubP)	Peptidergic trigeminal nerve fibers	Accurate YMC1021	E9381 H8247	rat; 1:500
Transient receptor channel M5 (TRPM5)	Taste transduction cascade	Dr. Emily Liman, University of Southern California, Los Angeles, CA	—	rabbit; 1:1000
Transient receptor channel M5 (TRPM5)	Taste transduction cascade	Abcam AB74849	786146	rabbit; 1:500
Vesicular acetylcholine transporter (VAChT)	Cholinergic cells	Sigma V5387	024K4774	rabbit; 1:200
Vesicular acetylcholine transporter (VAChT)	Cholinergic cells	Promega G448A	8302401	goat; 1:1,000

## Results

### Expression of non-olfactory markers in the main olfactory epithelium

**Fig 2:**
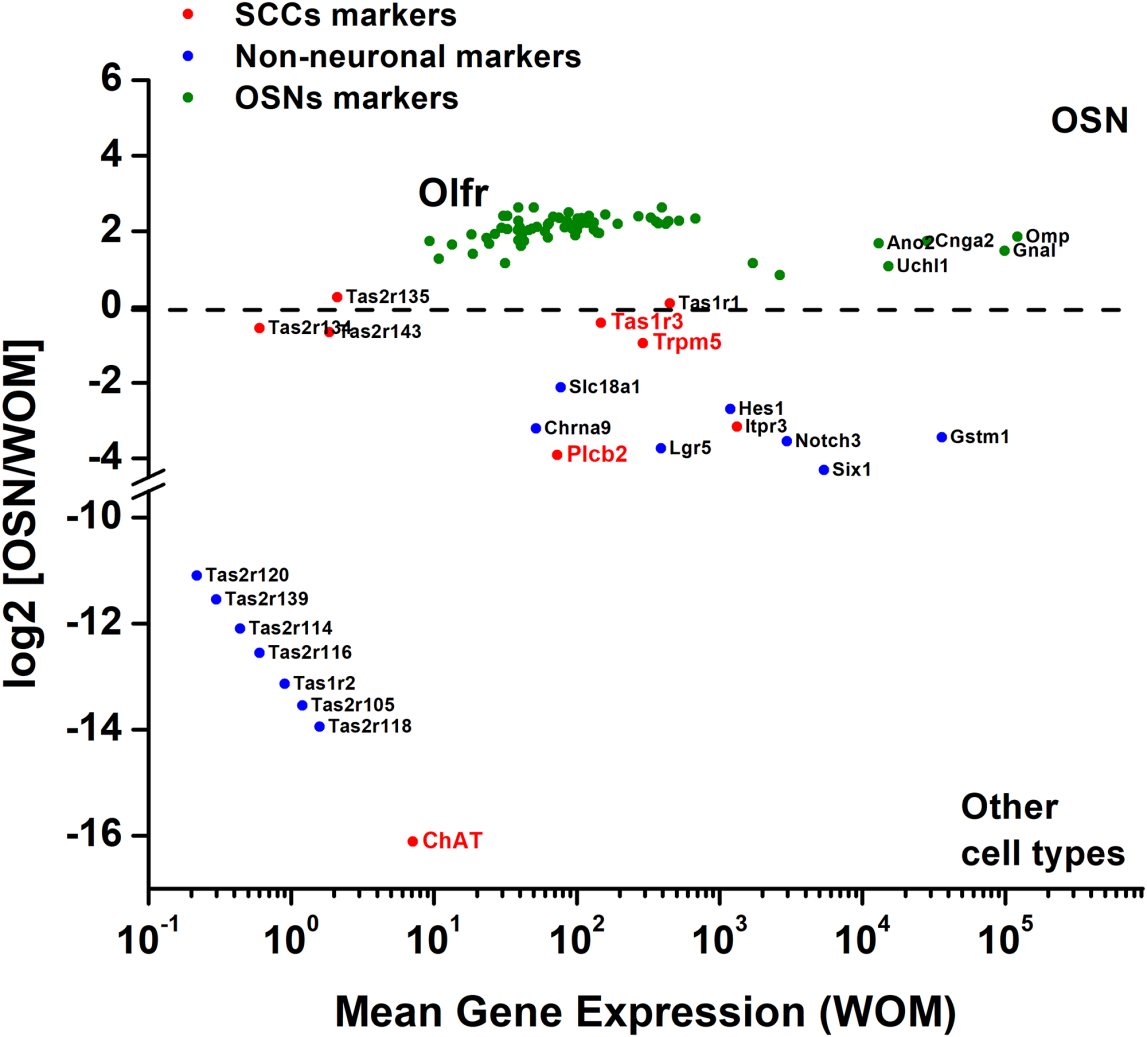
Differential gene expression analysis of mouse OSNs and whole olfactory mucosa (WOM). Fold-change was calculated as [OSNs]/[WOM], which gives positive results for those genes mainly expressed in the OSNs (green; Olfr, all olfactory receptors), while negative values can be associated to genes more abundant in non-neuronal cell types of the epithelium (blue and red). Underlined genes in red are the typically expressed by SCCs whose expression in MVCs was analyzed in this study.

We analyzed the RNA-seq data set of C57BL/6 mouse olfactory mucosa and single neuron transcriptomes (S1 Table) [21]. The differential expression of OSN-specific markers was compared to the elements of the bitter taste transduction signaling cascade. Fig 2 shows the average gene expression in the WOM in relation to the base 2 logarithm of fold-change ([OSN]/[WOM]). Genes with positive values above log_2_-fold change of gene expression, such as *Cnga2*, *Omp*, *Ano2*, and all the olfactory receptors (Fig 2, green) are highly expressed in the OSNs and relatively scarce or absent in other cells types of the WOM. Genes with negative values of log_2_-fold change can be mostly detected in cell types other than OSNs, such as *Lgr5*, a marker of the globose cells, or *Notch3*, known to be expressed in the supporting/sustentacular cells (Fig 2, blue). From the Saraiva dataset [21], we also analyzed *TrpM5* and *Chat*, which are known to be express in MVCs. We calculated that *TrpM5* is 0.95-fold higher in the WOM than in the OSNs (S1 Table), suggesting the expression of this gene mostly in non-neuronal cells, such as MVCs [19], and in a subset of OSNs as previously reported [17]. ChAT level of expression in the WOM is 16.12-fold higher than in the OSNs (S1 Table), consistent with specificity of this gene for only MVCs in the WOM, as previously described [27]. Similarly to SCC expression pattern, in the Saraiva RNA-seq data set other genes of the bitter taste transduction cascade could be detected in the olfactory mucosa, such as *Tas1r3*, *Plcb2*, and *Gnat3* (gustducin gene) (Fig 2, red). Saraiva’s data (summarized in S1 Table) detected *Tas1r3* RNA expression level in the WOM as mainly associated to the non-neuronal cell population, and as shown in the single OSN RNA-seq data, *Tas1r3* was occasionally found in OSNs. *Plcb2* and *Gnat3* expression patterns are more specific for mucosal cells other than OSNs [21]. Thus, while *TrpM5* and *Tas1r3* seem to be partially shared by both OSNs and MVCs, *Plcb2* and *Gnat3* are mainly expressed in non-neuronal Chat-positive MVCs.

### TRPM5-positive cells in the main olfactory epithelium

**Fig 3:**
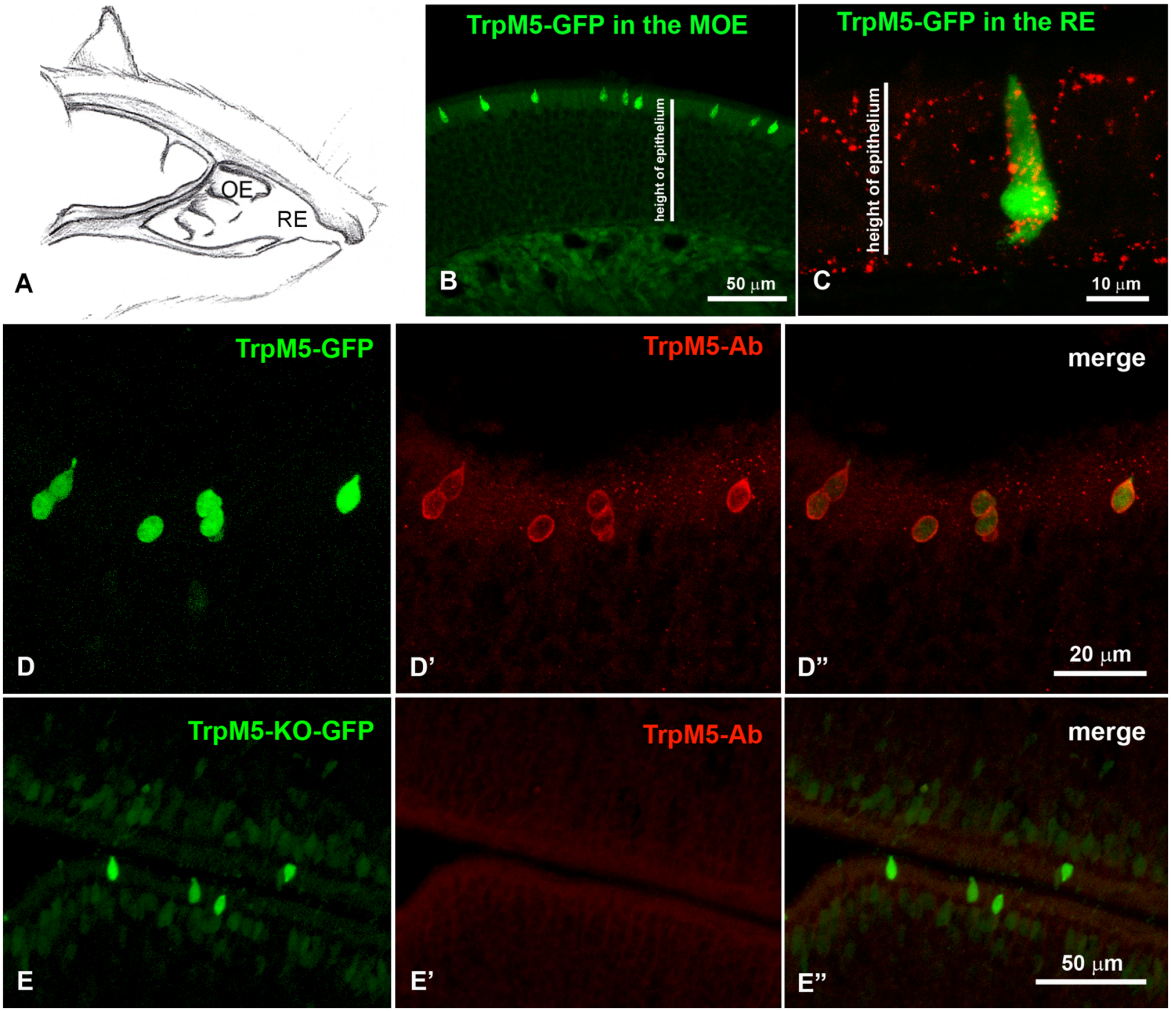
TRPM5-GFP-positive MVCs and validation of TRPM5 expression. **A**. Schematic representation of a mouse nasal cavity and the location of the olfactory (OE) and respiratory (RE) epithelia. **B**. TRPM5-GFP-positive MVCs in the MOE are located only in the uppermost portion of the epithelium. **C**. TRPM5-GFP-positive SCCs in the RE span for the whole depth of the epithelium. CGRP immunoreactivity (red) shows the intimate relation of SCCs with the trigeminal nerve fibers. **D-D′′**. Colocalization of TRPM5 antibody with GFP in MOE of TrpM5-GFP mice. **E-E′′**. TrpM5-GFP mice missing a functional *TrpM5* gene (TrpM5-GFP/TrpM5 knockout) were used to validate that the TRPM5 antibody labeling is absent in GFP-positive MVCs.

As reported previously, the TrpM5-GFP mouse nasal cavity (Fig 3A) contains two different non-neuronal cell types that express TRPM5: MVCs, located in the MOE with a short pear-shaped cell body (green, Fig 3B) [18,19,28], and SCCs in the RE with a long and narrow cell body spanning through the entire height of the RE and trigeminal innervation contacting them (Fig 3C, green and red, respectively) [12,13,15,29].

To confirm the expression of the TRPM5-channel in MVCs, cryosections of MOE from TrpM5-GFP mice and TrpM5-GFP/TrpM5-KO mice were immunostained with a custom-made TRPM5 antibody (kindly provided by Dr. Emily Liman, University of Southern California, Los Angeles, CA; see Table 1). In TrpM5-GFP mice, the antisera colocalized with the GFP signal (Fig 3D–3D′′), while in TrpM5-GFP/TrpM5-KO, no label colocalized with GFP-positive cells (Fig 3E–3E′′), demonstrating that the TRPM5 channel is specifically expressed in MVCs. A commercially available TRPM5 antiserum (Abcam; see Table 1) was also tested, showing a similar result.

### TRPM5-positive microvillous cells are not olfactory neurons

**Fig 4:**
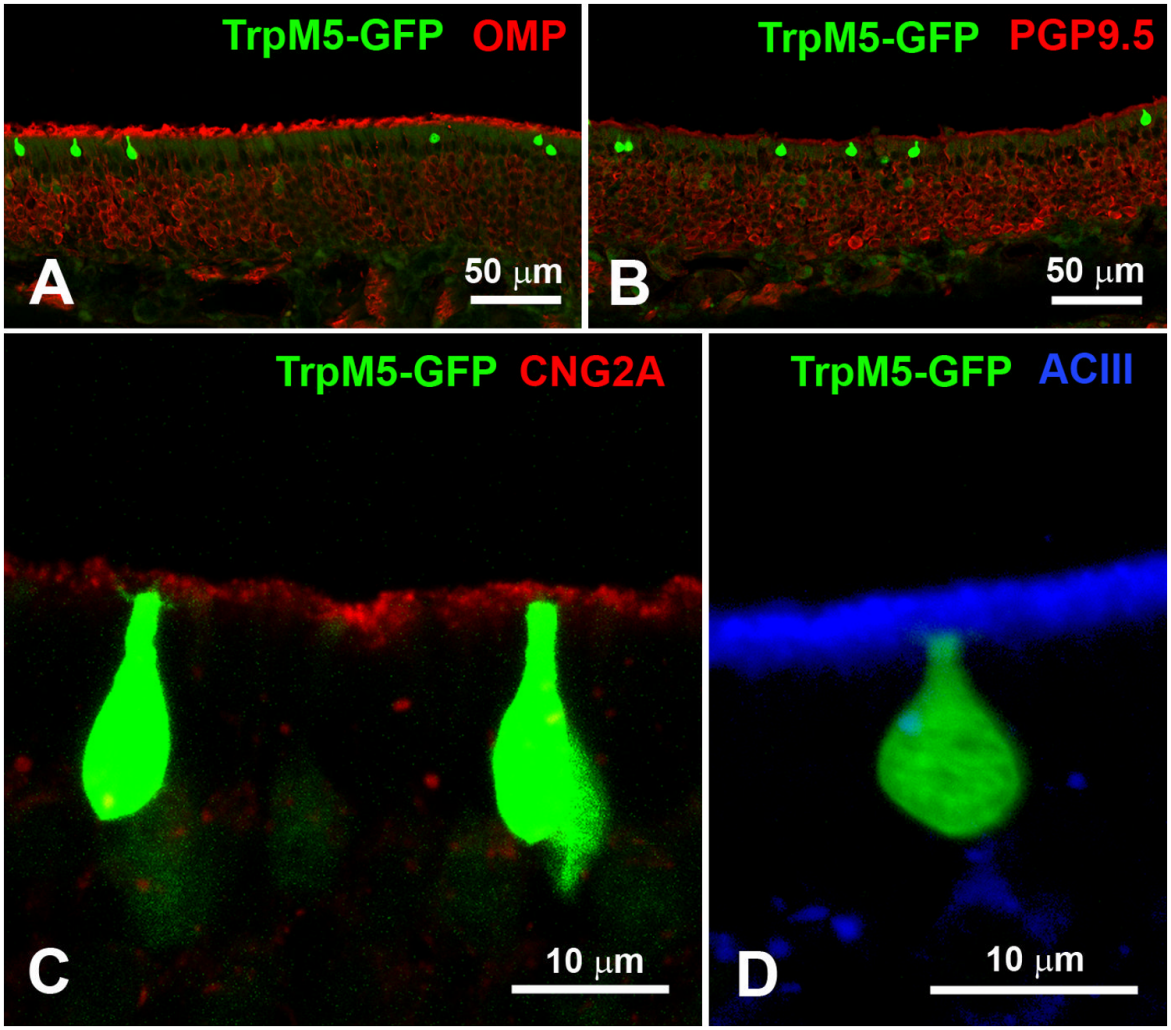
TRPM5-positive MVCs are not OSNs. **A and B**. OMP and PGP9.5, immunoreactive markers for OSNs, do not colocalize with GFP-positive MVCs in MOE from TrpM5-GFP mice. **C and D**. Immunoreactivity of CNG2A and ACIII, elements of the olfactory transduction signaling cascade, is not detected in TRPM5-GFP-positive MVCs.

The non-neuronal nature of TRPM5-positive MVCs in the MOE was verified by comparing GFP fluorescence in TrpM5-GFP mice and immunoreactivity for OMP (Fig 4A) and PGP9.5 (Fig 4B). We also tested two members of the olfactory transduction pathway: CNG2A (Fig 4C) and ACIII (Fig 4D; see Table 1 for antibodies description). Our immunostaining technique confirmed that neither OMP, PGP9.5, CNG2A, nor ACIII (Fig 4) co-localize with the TRPM5—GFPsignal. Therefore, TRPM5-positive cells located in the olfactory epithelium are not OSNs but MVCs, as previously reported [18,19].

### TRPM5-positive microvillous cells do express members of the taste transduction pathway

**Fig 5:**
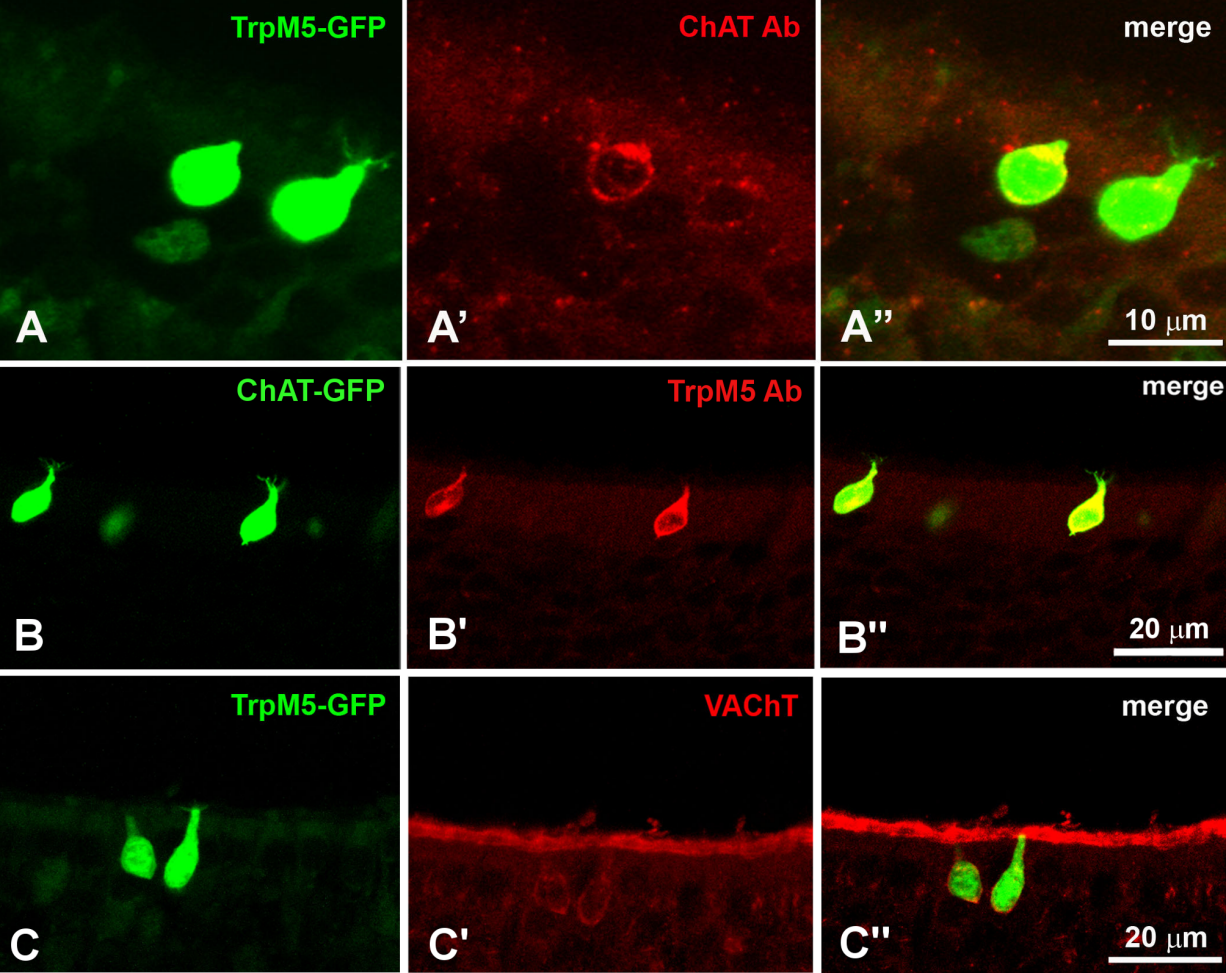
MVCs express markers of the cholinergic system. **A-A′′**. Antisera against ChAT labels TRPM5-GFP-positive MVCs. **B-B′′**. The antisera against TRPM5 colocalizes with the ChAT-GFP-positive MVCs. **C-C′′**. VAChT immunoreactivity in TRPM5-GFP-positive MVCs.

**Fig 6:**
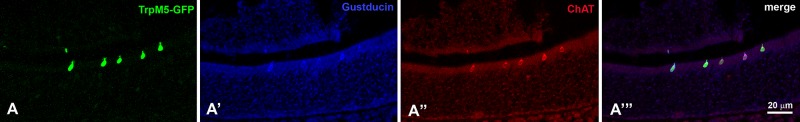
MVCs express elements of the taste transduction signaling cascade, similarly to SCCs. **A-A′′′**. Double staining with gustducin and ChAT of MOE of TrpM5-GFP mice. GFP-positive MVCs co-label with gustducin (in blue, A′) and ChAT (in red, A′′).

**Fig 7:**
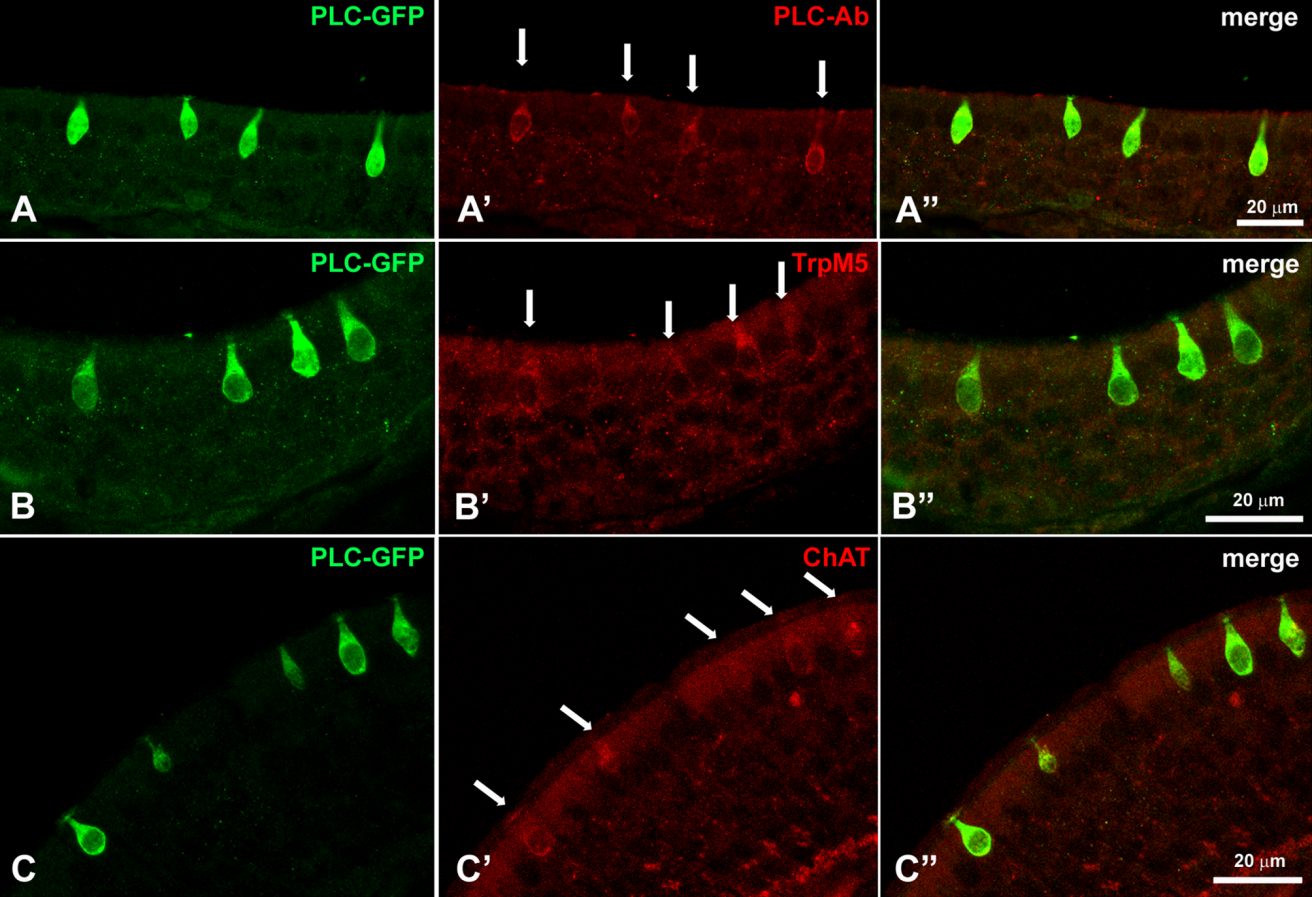
Plcb2-GFP mouse confirms the presence of PLCβ2 in MVCs, and Chat-GFP mouse validates the presence of elements of the cholinergic system in MVCs. **A-A′′**. The antiserum against PLCβ2 labels the PLCβ2-GFP-positive MVCs, but there are no GFP-positive OSNs. **B-B′′**. TRPM5 immunoreactivity co-labels the PLCβ2-GFP-positive MVCs. **C-C′′**. ChAT antibody colocalizes with PLCβ2-GFP-positive MVCs. White arrows indicate immunoreactive MVCs.

MVCs and SCCs share common markers, such as TRPM5, ChAT, and VAChT [20], but it was unclear if other SCC markers are also shared. Our staining for ChAT and VAChT (Table 1) on PLP-fixed samples confirmed the colocalization of these two markers in TRPM5-positive MVCs (Fig 5A–5A′′, 5B–5B′′ and 5C–5C′′). VAChT immunoreactivity was also visible in the superficial cell layer just below the cilia (Fig 5B′ and 5B′′). Gustducin, is an important component of the taste transduction signaling pathway known to be expressed in the SCCs, have not been previously reported in MVCs [18,19]. Here we show that gustducin antibodies on PLP-fixed tissue of TrpM5-GFP mice colocalize with ChAT and GFP (Fig 6). In the MOE, TRPM5/ChAT-positive MVCs showed gustducin immunoreactivity (Fig 6A–6A′′′), Considering that in the RNA-seq data Plcb2 is mostly expressed in non-neuronal cells, we analyzed the localization of PLCβ2 by taking advantage of the existing Plcb2-GFP mouse developed in the Chaudhari laboratory. MOE tissue from Plcb2-GFP mice was immunostained with antibodies for PLCβ2, TRPM5, ChAT(Fig 7). We observed GFP fluorescence only in the MVCs, as the only GFP-positive and therefore PLCβ2-expressing cells in the MOE. PLCβ2 antibody labeled all the GFP-positive cells in the Plcb2-GFP mouse without any staining in the OSN layer (Fig 7A–7A′′). The antiserum against TRPM5 that labeled TRPM5-positive MVCs (Fig 3D′ and 3E′) also labeled PLCβ2-positive MVCs in the Plcb2-GFP mouse (Fig 7B–7B′′). The antibody against ChAT also labeled the PLCβ2-GFP-positive MVCs (Fig 7C–7C′′).

**Fig 8:**
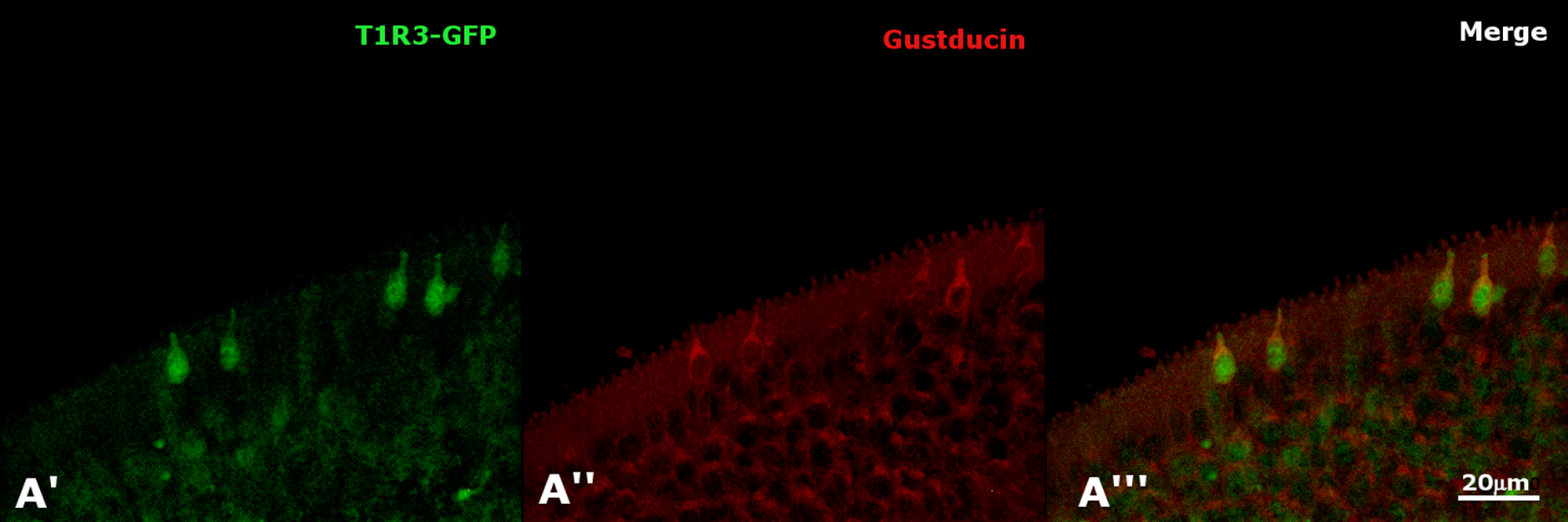
The taste receptor T1R3 is present in MVCs and possibly in OSNs as suggested by the RNA-seq data analysis. **A′-A′′′**. GFP-positive MVCs and few OSNs are visible in the MOE of Tas1R3-GFP mice, but only T1R3-GFP-positive MVCs colocalize with gustducin.

As previously shown in taste buds and SCCs, the G protein α-gustducin is coexpressed with the taste receptor T1R3 [11,30]; therefore, we investigated the possibility that MVCs could also express T1R3, as suggested by the RNA-seq data set. Using our transgenic Tas1R3-GFP mouse, we observed GFP-positive cells in the MOE possessing the same morphology of MVCs and verified the expression of gustducin in T1R3-GFP-positive cells demonstrating that MVCs express the taste receptor T1R3 (Fig 8).

### Sparse innervation of TRPM5-positive microvillous cells by trigeminal fibers

**Fig 9:**
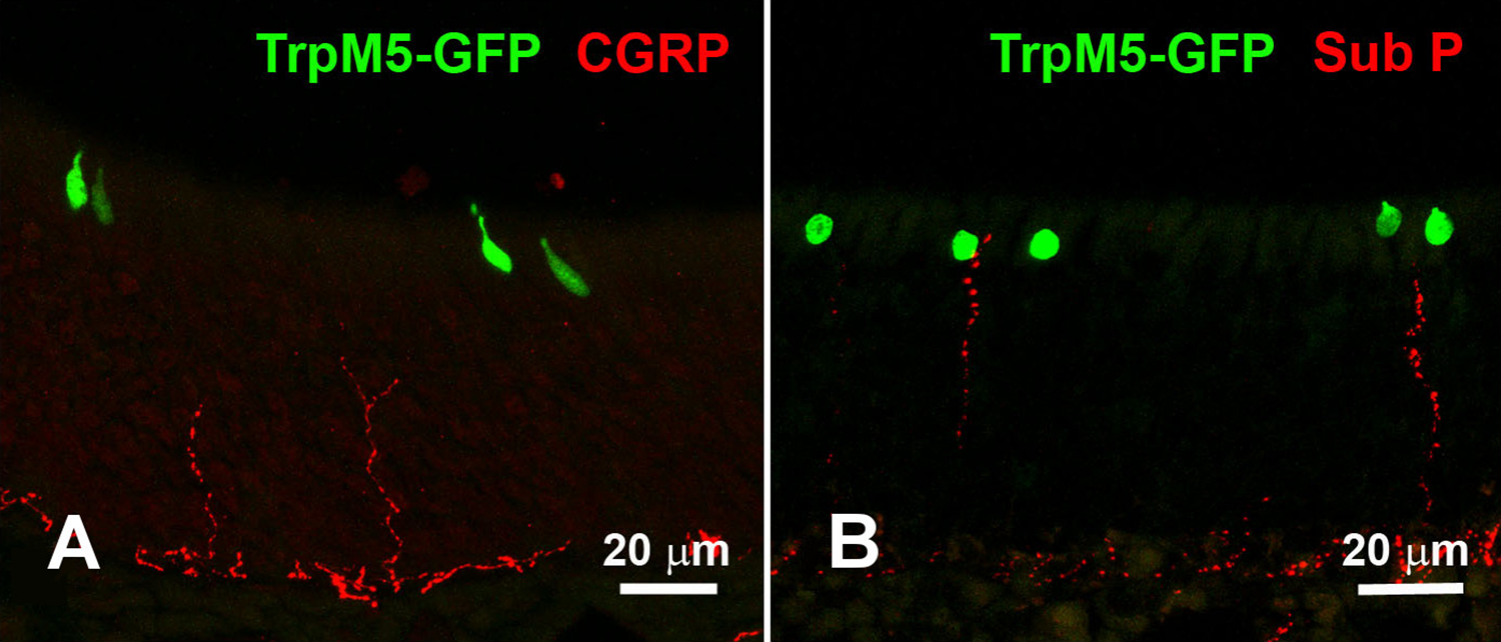
Peptidergic trigeminal nerve fibers rarely create a direct contact with MVCs. **A**. CGRP-immunoreactive trigeminal fibers seem to never contact TRPM5-GFP-positive MVCs. **B**. Occasionally, SubP-immunoreactive trigeminal nerve fibers in spatial proximity of TRPM5-GFP-positive MVCs.

Since the TrpM5-expressing SCCs in the RE are heavily innervated by peptidergic trigeminal nerve fibers [9,12], we tested PLP-fixed tissue with antibodies against the trigeminal neuropeptides CGRP and SubP. These neuropeptides are stored in vesicles along the axon of peptidergic trigeminal fibers [7], and as previously reported, we could not detect any contact between CGRP-positive nerve fibers and TRPM5-positive MVCs (Fig 9A) [18,19], with the exception of some rare spatial proximity that could occasionally be detected between TRPM5-positive MVCs and SubP-positive nerve fibers (Fig 9B), but never contact them as with the SCCs in the RE [12]. So, unlike SCCs, which are chemosensory cells with a trigeminal nerve partnership, MVCs in the MOE are chemoreceptor cells that modulate only the local epithelium, as previously reported [20].

## Discussion

Taking advantage of four genetically modified TrpM5-GFP, Plcb2-GFP, Chat-GFP, and Tas1R3-GFP mice, existing RNA-seq data of olfactory mucosa and single neurons transcriptomes [21], and a different tissue fixation procedure than the one previously used [18,19], we characterized the population of TRPM5-positive MVCs in the MOE. We determined that TRPM5/ChAT-positive MVCs coexpress the typical elements other of the taste transduction signaling and SCC cascades: the taste receptor T1R3, the G-protein α-gustducin, and the phospholipase PLCβ2. From staining and RNA-seq data we can conclude that MVCs express the molecular machinery typical for bitter and sweet taste transduction cascades (Fig 1; the SCC markers newly observed in MVCs are underlined).

The transduction signaling cascade composed of taste receptors, gustducin, PLCβ2, and TRPM5 is involved in the transduction of the taste responses [10,31–33], as well as in the detection of irritants and bacterial signaling molecules by the SCCs in the RE [9,12–14]. Although TRPM5-positive MVCs share similar molecular machinery with the SCCs, we can only speculate that MVCs could be able to detect irritants and bacterial compounds. Recently, it has been shown that MVCs can respond to denatonium and modulate the function of the neighboring supporting/sustentacular cells and OSNs, probably by releasing ACh [20]. Here we also show that, similarly to SCCs in the RE and in the vomeronasal organ duct and in the taste cells of taste buds [16,27,34], TRPM5-positive MVCs express the enzyme ChAT and VAChT. ChAT is necessary for the synthesis of ACh, which is then transported into vesicles by VAChT [35]. As shown for taste cells and SCCs, ACh can be released upon activation of the taste transduction cascade [16,36]. In the case of SCCs, the released ACh activates nicotinic-acetylcholine receptors on the trigeminal nerve fibers, triggering central nervous system–mediated protective reflexes (apnea) and inflammatory responses [16]. Differently from what was shown for SCCs, TRPM5-positive MVCs rarely make contact with SubP- or CGRP-positive trigeminal fibers (Fig 9) [9,13,16]. Therefore, MVCs probably modulate neighboring cells (sustentacular cells and OSNs) by releasing ACh, similar to what was previously reported for tracheal brush cells and MVCs in the OE [20,37]. In the MOE, sustentacular cells have properties similar to the glia in the central nervous system, providing metabolic and physical support to the OSNs [38]. Sustentacular cells are rich in smooth endoplasmic reticulum and enzymes and are able to metabolize xenobiotic substances [39–45], but it was also shown that these cells respond to ACh by increasing their intracellular Ca^2+^ concentration [20].

Our data suggest a chemosensory function for the TRPM5-positive MVCs, whose detection of xenobiotic agents would induce the activation of the taste transduction signaling cascade and consequent release of the neurotransmitter ACh to stimulate sustentacular cells and promote the catabolism of dangerous substances.

In conclusion, the mouse nasal cavity hosts two populations of cells, SCCs and MVCs that express elements of the taste transduction signaling (Fig 1). Both cell types express T1R3, gustducin, PLCβ2, TRPM5, ChAT, and VAChT, but their morphological differences and opposite innervation profile suggest a slightly different function. SCCs, located in the RE, have a more slender and bipolar morphology, have “soft” microvilli, and make contacts with peptidergic trigeminal nerve fibers. In contrast, MVCs are located in the MOE, are “pear shaped” with “stiff” microvilli, and do not appear to be innervated by peptidergic trigeminal nerve fibers. Whereas the activation of SCCs by irritants and bacterial molecules can trigger quick defensive responses, as coughing, apnea, reflexes, and inflammation, through the trigeminal system [9,14], the lack of the trigeminal innervation in MVCs would not allow for a quick defensive response, so MVCs probably assist in protecting OSN function by stimulating sustentacular cells in metabolizing and eliminating xenobiotic agents. We can conclude that MVCs are molecularly and functionally almost identical to SCCs but that their action might be limited to the protection of the local olfactory epithelium, whereas SCCs are involved in the protection of the entire upper airways.

## Supporting information

S1 Table Differential gene expression in the olfactory sensory neurons and whole olfactory mucosa for OSNs, SCCs and Non-neuronal markers. Analysis of differential gene expression of mouse olfactory sensory neurons (OSNs) and whole olfactory mucosa (WOM) obtained by Saraiva et al. [21]. The data reported in this table are the represented in Fig 2. Original data: https://doi.org/10.1038/srep18178.
(XLSX)

S1 File NC3Rs ARRIVE guidelines checklist. (PDF)
